# Tissue Culture Response of Ornamental and Medicinal *Aesculus* Species—A Review

**DOI:** 10.3390/plants11030277

**Published:** 2022-01-20

**Authors:** Snežana Zdravković-Korać, Jelena Milojević, Maja Belić, Dušica Ćalić

**Affiliations:** Department of Plant Physiology, Institute for Biological Research “Siniša Stanković”, University of Belgrade, 11060 Belgrade, Serbia; jelena.milojevic@ibiss.bg.ac.rs (J.M.); mmilic0905@gmail.com (M.B.); calic@ibiss.bg.ac.rs (D.Ć.)

**Keywords:** aescin, androgenesis, *Aesculus*, genetic transformation, hairy roots, shoot organogenesis, somatic embryogenesis, secondary somatic embryogenesis

## Abstract

Species of the genus *Aesculus* are very attractive woody ornamentals. Their organs contain numerous health-promoting phytochemicals. The most valuable of them—aescin—is used in commercial preparations for the treatment of venous insufficiency. The industrial source of aescin is horse chestnut seeds because the zygotic embryos are the main site of its accumulation. Horse chestnut somatic and zygotic embryos contain similar amount of aescin, hence somatic embryos could be exploited as an alternative source of aescin. Somatic embryogenesis, androgenesis and de novo shoot organogenesis were successfully achieved in several *Aesculus* species, as well as secondary somatic embryogenesis and shoot organogenesis, which enables mass production of embryos and shoots. In addition, an efficient method for cryopreservation of embryogenic tissue was established, assuring constant availability of the plant material. The developed methods are suitable for clonal propagation of elite specimens selected as the best aescin producers, the most attractive ornamentals or plants resistant to pests and diseases. These methods are also useful for molecular breeding purposes. Thus, in this review, the medicinal uses and a comprehensive survey of in vitro propagation methods established for *Aesculus* species, as well as the feasibility of in vitro production of aescin, are presented and discussed.

## 1. Introduction

Species of the genus *Aesculus* are among the most attractive ornamental trees or shrubs, frequently used in urban greening and landscape architecture [1]. The genus *Aesculus* (*Hippocastanaceae*) comprises 12 species organized into five sections, based on morphological characteristics and molecular markers [2]. Since *Aesculus* species could cross in nature and were cultivated and bred by humans for decades, a number of varieties, cultivars and interspecific hybrids are available [1], such as *A. carnea* [3] and *A. x arnoldiana* [4]. These species are widely distributed throughout the temperate regions of the northern hemisphere, with seven species found in North America, one specie in Europe and five species in Asia. Among the *Aesculus* species, *A. hippocastanum* has the widest areal. It is native to the Balkan Peninsula, but it was introduced to urban areas of central Europe in the 17th century and subsequently spread throughout the temperate zone of the northern hemisphere [5].

The horse chestnut trees are tolerant to pollution in the urban environment [6,7], moderately susceptible to powdery mildew, anthracnose and leaf blotch, but very susceptible to the leafminer *Cameraria ohridella* [1,8]. *Aesculus* species exhibit different levels of susceptibility to the leafminer, with horse chestnut being the most susceptible species and the main host of the moth [9]. After its initial discovery in Macedonia in 1984 [8], *C. ohridella* infestation has spread across Europe over the last four decades and heavily damaged the horse chestnut plants [10,11,12]. Due to the activity of the leafminer, horse chestnut trees suffer from severe leaf damage, causing precocious defoliation, decreasing nutrient accumulation and seed weight, and affecting growth of the seedlings [13,14]. High variation in resistance to the moth was also found among the specimens within the species [15], presumably due to variable content of phenolic and saponin compounds in the leaves [16,17,18,19].

Apart from ornamental purposes, horse chestnut seeds are exploited by the pharmaceutical industry as an industrial source of aescin, used in commercial preparations for the treatment of venous insufficiency [20,21]. Horse chestnut seeds are also used for preparation of starch-based materials [22], as well as for the production of alcohol, dyes, and, to a lesser extent, in carpentry and woodcarving [23].

Tissue culture methods enable mass production of consistent plant material, which could be further used for the production of standardized-quality phytopharmaceuticals [24] and for commercial production of planting material [25]. In woody plant species, induction of in vitro regeneration is rather difficult because the plants become more recalcitrant to in vitro regeneration as they lose juvenility [26]. Since juvenility exerts a profound impact on the ability of explants to be successfully propagated in vitro, juvenile tissues of the adult trees are used for culture initiation [26]. Immature zygotic embryos usually exhibit the highest responsiveness in tissue culture, hence these explants have been most frequently used for culture initiation in woody plants [26]. However, zygotic embryos are not genetically identical to the parent and thus not suitable for clonal propagation of the selected elite specimens. Therefore, other juvenile tissues or organs have been used for this purpose. In *Aesculus* species, numerous procedures for in vitro regeneration have been developed, starting from various explants and using different regeneration pathways, including somatic embryogenesis, androgenesis and de novo shoot organogenesis.

To date, ethnobotanical uses, chemical structure and pharmacological activities of extracts obtained from *Aesculus* plants were reviewed in exhaustive studies by Zhang et al. [27] and Idris et al. [28], while the initial achievements in in vitro regeneration of horse chestnut were reviewed by Radojević [23,29] and Gastaldo et al. [30]. A plethora of findings that were reported subsequently prompted us to compile this review, in which a short overview of the recent achievements in medicinal uses of *Aesculus* species and a comprehensive survey of in vitro propagation methods established for these species will be presented and discussed.

## 2. Medicinal Uses of *Aesculus* sp.

All the plant parts of all *Aesculus* species, including seeds, seedlings, leaves, bark, flowers and even honey from the *Aesculus* flowers are poisonous. In spite of this, all members of the genus *Aesculus* have traditional and medicinal uses. Since ancient times, local populations in different parts of the world have been using various plant extracts, mainly the seed extract, for healing numerous medical problems. This is due to the fact that organs of these plants contain more than 200 valuable phytochemicals, including triterpenoids, triterpenoid glycosides (saponins), flavonoids, coumarins, carotenoids, long-chain fatty acid compounds [27], etc. Among them, aescin is the most valued and widely used in commercial preparations, mainly for the treatment of chronic venous insufficiency, hemorrhoids, post-operative edema [20,21,31,32] and as a remedy for cellulitis in the cosmetics industry [33]. Commercial preparations of aescin are available as oral tinctures, tablets and gels.

Aescin is a mixture of chemically related triterpenic glycosides. It is widely present in the seeds of virtually all *Aesculus* species: *A. assamica* [34,35,36], *A. californica* [37], *A. chinensis* [38,39], *A. glabra* [40], *A. hippocastanum* [41,42], *A. indica* [41], *A. pavia* [43,44] and *A. turbinata* [45,46]. Aescin content, in terms of compound profile and quantity, varies among the *Aesculus* species [44,47]. However, only *A. hippocastanum* and *A. chinensis* are officially recognized for medical uses to date [27].

Many biological activities of aescin validate the traditional use of seed extracts. Aescin exhibits potent anti-inflammatory [31,32,48,49], anticancer [44,50,51,52], antiviral [46,53,54,55], antifungal [35], antidiabetic [34,45,48], antiedema [32], antioxidative [49,56,57] and antiapoptotic [39] effects. Aescin is a promising phytochemical for anticancer drug development [50], as well as for the treatment of neurodegenerative diseases [39], neuropathy [58] and diabetic nephropathy [59]. Since it can reduce inflammation and edema, aescin is being considered for use in addon therapy in acute lung injury related to COVID-19 infection [60]. Furthermore, saponins, including aescin, are a promising new general drug delivery system for cytosolically active macromolecules [51,61]. Due to their ability to stabilize emulsions and foams and to solubilize hydrophobic molecules, saponins are also used in cosmetic and food products [62].

Currently, horse chestnut seeds are the sole industrial source of aescin. Within the seeds, aescin is present only in the zygotic embryos, which constitute only a small portion of the seed and are available only briefly during the year. Aescin content in horse chestnut seeds is highly variable depending on genotype, tree location (0.82–4.16%) [63], seed maturity (2.41–8.29%) [42] and seed storage [64]. It depends on the environmental conditions, but also varies in the seeds of the same genotypes over time [65].

## 3. Morphogenesis In Vitro

Thus far, numerous procedures have been developed for in vitro regeneration of *Aesculus* species. Various explant types were used for culture initiation for somatic embryogenesis or de novo shoot organogenesis induction. Embryo regeneration from both anther culture and microspore suspensions enabled the production of haploid plants [23,29]. However, in vitro regeneration techniques were established in only a few *Aesculus* species: *A. hippocastanum*, *A. flava* and *A. glabra*, and hybrids *A. carnea* and *A. x arnoldiana.* A survey of the methods used for in vitro regeneration will be presented in detail in the following sections.

### 3.1. Somatic Embryogenesis

Somatic embryogenesis is the process of reprogramming somatic cells from gametophytic to sporophytic, i.e., the embryogenic pathway of development, resulting in embryo formation. Thus, somatic embryos (SEs) are genetically identical to the parent plant. Because of this, somatic embryogenesis has been recognized as a superior method for clonal propagation of elite specimens, especially in woody plant species [25,66,67,68]. Bearing in mind that the embryogenic tissue is an ideal material for cryopreservation [69,70,71,72,73], this is the method of choice for the maintenance of juvenile plant material during field-testing of clonal lines, as well as for clonal propagation of previously selected and genetically tested elite tree specimens [74,75].

*Aesculus* species are prone to somatic embryogenesis, thus it is not surprising that somatic embryogenesis was successfully achieved with almost all horse chestnut plant organs used as initial explants: stamen filaments [76,77,78], immature zygotic embryos [77,79], seedling’s primary leaves [80] and cotyledons [81], and stem [82] and bark fragments [83] isolated from young terminal branches of adult trees. Somatic embryogenesis was also initiated from stems [84] and shoots [85] of *A. glabra*, stamen filaments of *A. flava* [86] and *A. carnea* [87], shoots and roots of 4-week-old seedlings and shoots of a 30-year-old tree of a hybrid *A. x arnoldiana* [85]. In all the studies, somatic embryogenesis was induced indirectly through a callus phase, with the exception of stamen filaments of *A. hippocastanum*, in which SEs regenerated directly from filaments in 10% of the explants, while in the remaining 90% of the explants SEs regenerated indirectly from embryogenic callus [77].

#### 3.1.1. Initiation of Embryogenic Cultures

For callus induction, various explants differed in their requirements for plant growth regulators (PGR). Auxins were found indispensable for embryogenic callus induction for most explants. In *A. hippocastanum*, 2,4-dichlorophenoxyacetic acid (2,4-D) was used for callus initiation in all following studies. Callus was induced from a seedling’s primary leaves, stem and bark fragments using the same PGR combination: 9.3 μM 6-furfurylaminopurine (Kinetin, Kin) + 10.7 μM α-naphthaleneacetic acid (NAA) + 9 μM 2,4-D [80,82,83]. For callus induction from cotyledons, only 0.45 μM 2,4-D was sufficient [81], while for immature zygotic embryos 13.6 μM 2,4-D + 4.6 μM Kin [79] or 4.4–8.8 μM 2,4-D + 5.4 μM NAA [77] were used. However, in most of the studies no comparative analysis of a range of PGR concentrations and types was conducted, and SE initiation frequencies were not reported, thus PGR combinations may be further optimized for increased embryogenic response. For callus induction from horse chestnut stamen filaments, Jörgensen [76] used several combinations of 2,4-D (0, 1, 2.5 or 5 μM) with 6-benzyladenine (BA at 0 or 5 μM) and found that each containing 2,4-D above 1 μM was effective. Later on, Capuana and Debergh [88] and Capuana [78] used 9 μM 2,4-D for callus induction from these explants. Summary of the literature data on somatic embryogenesis induction in *Aesculus* species is given in Table 1.

**Figure 1:**
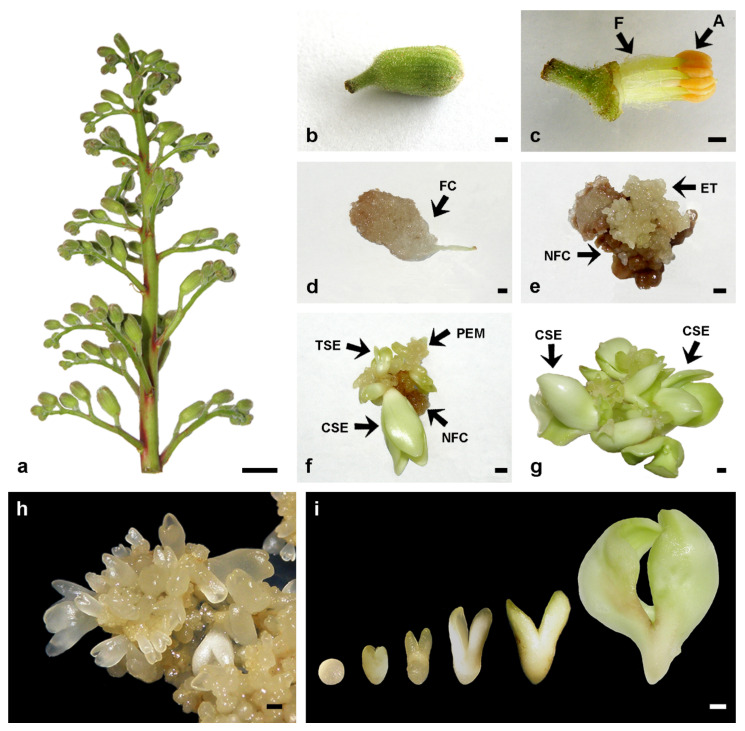
Induction of somatic embryogenesis from stamen filaments of *Aesculus flava*. (**a**) Inflorescence with closed flower buds, suitable for flower bud isolation. (**b**) A 7 mm-long flower bud selected for filament isolation. (**c**) The perianth was removed from the surface-sterilized flower bud, the anthers (A) were discarded, and the filaments (F) used for culture initiation. (**d**) Friable callus (FC) formation from a filament cultivated on callus induction medium (CIM) supplemented with 1 μM 2,4-D + 10 μM Kin in darkness for eight weeks. (**e**) Embryogenic tissue (ET) formation on the surface of necrotic friable callus (NFC) from the filaments cultivated on CIM for 8 weeks and then on plant growth regulator (PGR)-free MS medium for an additional 2-week period. (**f**) Somatic embryo (SE) regeneration from proembryogenic masses (PEM) after a 4-week-cultivation on PGR-free medium. Arrows indicate SEs at torpedo (TSE) and cotyledonary (CSE) stages of development. (**g**) SEs multiplied by secondary somatic embryogenesis on PGR-free medium. Numerous SEs reached the cotyledonary stage (CSE) of development after 4–8 weeks of cultivation on PGR-free medium. (**h**) PEM and SEs were maintained through repetitive cycles of secondary somatic embryogenesis on MS medium supplemented with 0.05 μM 2,4-D + 5 μM Kin + 400 mg/L glutamine. (**i**) SEs at all stages of development, from the globular to the late cotyledonary stage of development were observed. Scale bars: 1 cm (**a**); 1 mm (**b**–**i**). Unpublished from the authors’ Laboratory.

In *A.* *flava*, combinations of 1, 5 or 10 μM 2,4-D with 0, 1, 5 or 10 μM Kin were tested in order to determine the most efficient combination of these PGRs for callus induction and subsequent SE regeneration from filament explants [86]. In contrast to horse chestnut filaments [78,88], 2,4-D as a sole PGR was not sufficient to trigger efficient callus induction in *A. flava* [86]. At the highest tested concentration, 10 μM 2,4-D without Kin, only 36% of the explants produced small calli (that remained non-embryogenic throughout the duration of the experiment). For all 2,4-D/Kin combinations, the frequencies of callus formation reached 100%. However, the highest callus yield, SE regeneration frequency and mean SE number per explant were attained when the filament explants were cultivated on medium supplemented with 1 μM 2,4-D + 10 μM Kin [86]. The most important steps in SE induction from filaments of *A. flava* are shown in Figure 1.

In *A. x arnoldiana*, BA (at 5 or 25 μM) as a sole PGR was sufficient for SE induction from shoot and root explants of 4-week-old in vitro seedlings and shoots from a 30-year-old tree. Shoots of *A. x arnoldiana* in vitro seedlings exhibited significantly higher SE regeneration frequency (53%) than the root explants (22%) [85].

BA at 5 or 25 μM was also used to induce embryogenic calli from shoot explants of 3-year-old plants of *A. glabra*, with regeneration frequencies 19% and 42% with 5 μM and 25 μM BA, respectively [85]. However, Trick and Finer [84] found both 2,4-D and Kin indispensable for induction of embryogenic calli from stem explants of 3-week-old seedlings of *A. glabra*. The frequencies of embryogenic callus formation were low: 2.8% and 8.3% in explants cultivated on MS medium supplemented with 4.5 μM 2,4-D + 4.7 μM Kin or 9.1 μM 2,4-D + 9.3 μM Kin, respectively [84]. The leaf blade fragments and petioles produced no SEs under these conditions.

Generally, juvenile tissues exhibited better embryogenic response. Accordingly, shoots isolated from 4-week-old in vitro seedlings of *A. x arnoldiana* exhibited significantly higher SE regeneration frequency (41%) than shoots isolated from cuttings of a 30-year-old tree (3%) when cultivated in the light [85]. Interestingly, shoots isolated from 3-year-old plants of *A. glabra*, a parent of *A. x arnoldiana*, showed intermediate SE initiation frequency (19%) under the same experimental conditions [85]. However, the observed differences may also be due to the use of different genotypes as sources of explants. In line with the aforementioned, seedling organs [79,80,81,84,85] or juvenile tissues of adult trees [76,82,83,86] were successfully used for SE initiation. Thus, very old (100 years) elite specimens could be cloned using some of these methods [76].

Media for cultivation of various explants taken from *Aesculus* sp. contained Murashige and Skoog mineral solution (MS) [89] in the majority of studies [77,78,79,80,81,82,83,84,86], while Woody Plant Medium (WPM) [90] was used in much fewer studies [76,85]. In some studies, additional adjuvants were added to the media for embryogenic callus induction, most frequently amino acids: glutamine, glycine, serine [76] and proline [79], as well as casein hydrolysate [79,86].

In the majority of studies, explants were exposed to light conditions from culture initiation, commonly to a 12 h- or 16 h-photoperiod and a photosynthetic photon flux density ranging from 30 to 110 μmol s^−1^ m^−2^. However, there are a few exceptions [77,85,86,88]. In *A. x arnoldiana* and *A. glabra*, regeneration frequencies were always significantly higher in the explants cultivated in darkness as compared to those cultivated in light [85]. In *A. flava*, light inhibited callus formation and SE regeneration from stamen filaments [86]. A minimum of four weeks of cultivation of the explants in darkness was necessary for the initiation of embryogenic callus, while 8–10 weeks of cultivation in darkness contributed to the highest embryogenic response of the explants.

Chilling was also reported to influence embryogenic capacity of the explants. In *A. glabra*, shoots isolated from 3-year-old plants were subjected to cold storage at 5 °C in darkness for 8 weeks, 8 weeks after initiation, and then returned to the light, which resulted in significantly higher SE initiation frequency (42%) compared to explants cultured continuously under light conditions at 25 °C (19%) [85]. By contrast, chilling of flower buds had an adverse effect on SE induction from stamen filaments of *A. hippocastanum* [77].

In most of the studies two types of calli were observed: non-embryogenic, white, compact calli and embryogenic, yellow, nodulated, friable calli (Figure 1d,e) [76,79,80,82,83,84]. Histological analysis revealed that whitish and compact non-embryogenic calli consisted of small and large parenchyma cells, while embryogenic calli produced clusters of proembryoids [91]. An ultrastructural study shed further light on the features of the cells found in the two types of calli. In non-embryogenic calli, intercellular spaces were scarce, and within the cells one huge vacuole and numerous autophagic vacuoles were observed; the mitochondria contained a few cristae, and the plastids contained no starch [92]. On the contrary, embryogenic cells had features typical of highly metabolically active cells: the ribosomes were grouped in polysomes, the mitochondria were rich in cristae, and numerous starch grains were seen in the plastids [92]. The embryogenic aggregates, observed within the embryogenic calli, contained small cells with large nuclei, dense cytoplasm, and plastids with numerous starch grains, which Profumo et al. [92] considered as embryoid initials.

#### 3.1.2. Proliferation of Embryogenic Cultures and Embryo Differentiation

Regardless of the explant type used for embryogenic callus induction, these calli looked similar—yellow, nodulated and friable [76,79,80,82,84,85,86]—and all gave rise to proembryogenic masses (PEMs). PEMs and SEs usually formed on embryogenic calli while still cultivated on callus induction medium (Figure 1e) [79,86], but for mass production the embryogenic calli should be subcultivated onto a medium devoid of (Figure 1f,g) or with lower levels of auxin [79]. In all the studies reported here, embryogenic calli were subcultivated either onto PGR-free medium [78,80,82,83,84,85,86,88] for further embryo development or on medium supplemented with 2.5 μM BA [76]. PEM formation from the juvenile explants was usually observed after 6–8 weeks of culture [80,85,86], while in explants taken from 3-year-old plants of *A. glabra* and a 30-year-old tree of *A. x arnoldiana* PEMs appeared 16–17 weeks after culture initiation [85].

Embryogenic tissues continued to proliferate on PGR-free medium for months or years [78]. However, for longer periods of time it is highly recommended that the embryogenic tissue is transferred to a medium containing cytokinins, e.g., 4.4 μM BA [78] or 0.05 μM 2,4-D + 5 μM Kin (Figure 1h) [86].

In all the studies mentioned above, asynchronous development of SEs (Figure 1f) and SEs at all stages of development (Figure 1i) were observed, along with irregularities in embryo anatomy, e.g., more than two cotyledons and/or cup- or funnel-shaped cotyledons, as well as physiological disorders such as albinism and hyperhydricity [78,79,86].

### 3.2. Androgenesis

Androgenesis and gynogenesis are special cases of embryogenesis because embryos originate from immature gametic cells and thus contain a half of chromosome number, giving rise to haploid plants. Haploids themselves are important for dihaploid production, because dihaploids contain two identical chromosome complements and represent pure lines suitable for breeding purposes [93,94]. Therefore, “haploids are sporophytic plants with the gametophytic chromosome number, while doubled haploids are haploid plants that underwent spontaneous or induced chromosome duplication” [95]. Instead of time-consuming and costly classical breeding, dihaploids are obtained in just two steps: the induction of haploid embryos and subsequent chromosome doubling [93,94,95]. This is especially important for woody plant species, which are characterized by a long juvenile phase and a high level of heterozygosity [95,96]. In haploids and dihaploids, recessive traits are fixed, thus these plants are a broad base for selection of new traits.

Androgenesis was achieved in several *Aesculus* species: *A. hippocastanum* [97,98,99], *A. carnea* [100,101] and *A. flava* [102,103]. All these studies were conducted in the authors’ laboratory.

**Figure 2:**
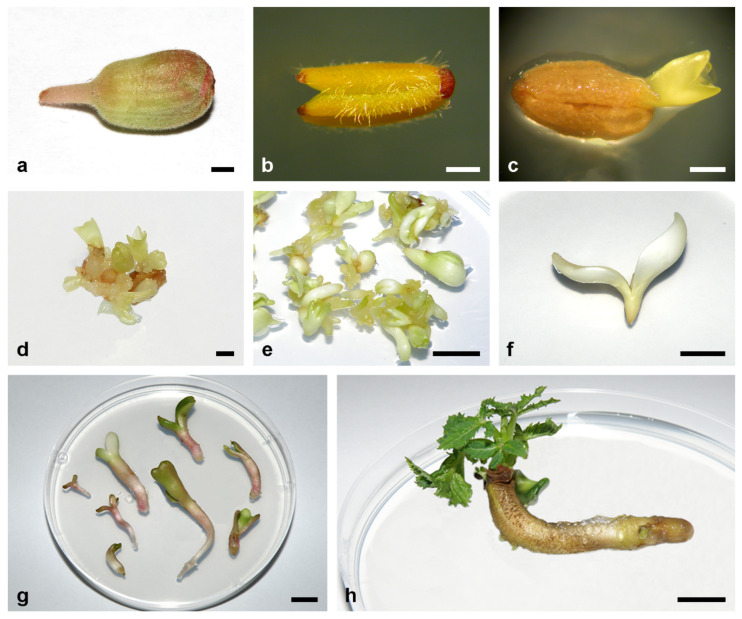
Induction of androgenesis in anther culture of *A. carnea*. (**a**) A 5 mm-long closed flower bud used for anthers isolation. (**b**) An isolated anther. (**c**) An embryo protruding from the anther cultivated on callus induction medium supplemented with 4.5 μM 2, 4-D + 4.6 μM Kin in darkness for 8 weeks and then on plant growth regulator-free MS medium supplemented with 400 mg L^−1^ of glutamine for 2 weeks. (**d**) Proembryogenic masses and embryos at different stages of development regenerated from the anther depicted in (**c**) two weeks later. (**e**) Embryogenic culture maintenance through secondary somatic embryogenesis on MS medium containing 0.045 μM 2,4-D + 4.6 μM Kin. (**f**) An albino embryo at the cotyledonary stage of development. (**g**) Embryos at the cotyledonary stage of development (~1 cm long) germinated on MS medium supplemented with 0.045 μM 2,4-D + 4.6 μM Kin for four weeks. (**h**) An embryo that germinated on MS medium supplemented with 0.045 μM 2,4-D + 4.6 μM Kin developed plumule after cultivation on MS medium supplemented with 5 μM Kin for an additional four weeks. Scale bars: 1 mm (**a**–**d**); 1 cm (**e**–**h**). Unpublished from the authors’ Laboratory.

Two types of cultures were used for induction of androgenesis in *Aesculus* species: anther culture and microspore suspension. Anther culture is easier to manipulate, but during the regeneration process both androgenic and somatic embryos develop. Androgenic embryos (AEs) regenerate from microspores, while SEs may regenerate from anther wall, tapetum cells or remains of filament and connective tissues [23]. Thus, in anther culture embryos of different ploidy levels can be obtained [98]. On the other hand, microspore culture enables production of haploids only, since somatic tissue is not present in the suspension due to sieving of cells through a filter that allows only microspores to pass through [98]. Summary of the literature data on androgenesis induction in *Aesculus* species is given in Table 2, and the most important steps in AE induction from anthers of *A. carnea* are shown in Figure 2.

#### 3.2.1. Anther Culture

The most important factors that impacted the efficiency of androgenic response were the stage of microspore development, genotype, media content and temperature of the environment in which donor plants were located several days prior to inflorescence harvest [97,99,101]. From the first report on androgenesis induction from anthers of horse chestnut, it became clear that the stage of microspore development had a decisive impact on regeneration success [97]. Generally, there is a narrow time window during microsporogenesis when the microspores are amenable to shift from gametophytic to sporophytic development. In horse chestnut, the microspores at the uninuclear stage of development are suitable for acquiring competence for embryogenesis [23,29,97]. Flower bud length proved to be a good visual marker for the stage of microspore development. The majority of anthers isolated from 4–7 mm flower buds of *A. hippocastanum* and *A. carnea* contained uninuclear microspores [97,101]. In both species the highest embryogenic response, 52.7% in *A. hippocastanum* and 22.4% in *A. carnea*, was obtained with anthers isolated from 4–5 mm flower buds [97,101], while only 2% of anthers isolated from horse chestnut flower buds longer than 7 mm were embryogenic [97].

2,4-D was indispensable for AE induction in horse chestnut [97], and MS medium supplemented with 4.5 μM 2,4-D + 4.6 μM Kin was very efficient for the induction of androgenesis [97]. The same 2,4-D/Kin combination, among several tested, gave the best results in *A. carnea* [100,101] and was also successfully used for the induction of embryogenesis from anthers of *A. flava* [102,103] and microspore suspensions of *A. hippocastanum* [98,99] and *A. flava* [103] (Table 2).

Frequency of androgenesis was highly dependent on genotype and varied between 5% and 37.6% in *A. hippocastanum* [99] and 1–38% in *A. carnea* [100,101] under the same experimental conditions (5 mm flower buds cultivated on MS medium supplemented with 4.5 μM 2,4-D + 4.6 μM Kin). Environmental temperature of 4–5 °C seven days prior to inflorescence harvest from the donor plants was optimal for androgenesis induction in *A. hippocastanum* [99].

Under cytological examination, divisions of the horse chestnut microspore nucleus into a large vegetative and a smaller generative nucleus were observed after one week of cultivation [23,97]. Later on, the generative nucleus degenerated, thus it was proposed that AEs arise only from the vegetative cell. After three weeks of anther culture, globular embryos were observed in the anther cavity. Cells of AEs at the globular stage of development were rich in organelles, showing high metabolic activity [104]. The embryos were observed with the naked eye 5–8 weeks after culture initiation [97,99].

AEs efficiently multiplied on PGR-free medium, but for long-term maintenance MS medium supplemented with 0.045 μM 2,4-D + 4.6 μM Kin (Figure 2e) was successfully used for all three species [98,99,100,101,102,103].

#### 3.2.2. Microspore Suspension Culture

For microspore suspension initiation, the anthers were macerated and suspended in liquid MS medium supplemented with 4.5 μM 2,4-D + 4.6 μM Kin, then the microspores were sieved through a 50-μm mash and shaken on a platform shaker at 85 rpm in darkness [98,99,103]. After 8 weeks of culture, the obtained suspensions were plated on a solid MS medium with 0.045 μM 2,4-D + 4.6 μM Kin.

Microspore culture proved to be a more efficient method for haploid production compared to anther culture [98,99,103]. Approximately three-fold more embryos and five-fold more green embryos per anther were obtained from microspore suspension culture in *A. hippocastanum* compared to anther culture [98]. In *A. flava*, 36.6% of anthers were embryogenic, while in microspore suspension 111.7 embryogenic clusters per suspension (made from 20 anthers) were obtained [103]. From the same amount of initial plant material (60 anthers), 333 AEs were obtained by anther culture, while 510 AEs were obtained using microspore suspension culture [103] after 16 weeks.

#### 3.2.3. Ploidy Level Determination

**Figure 3:**
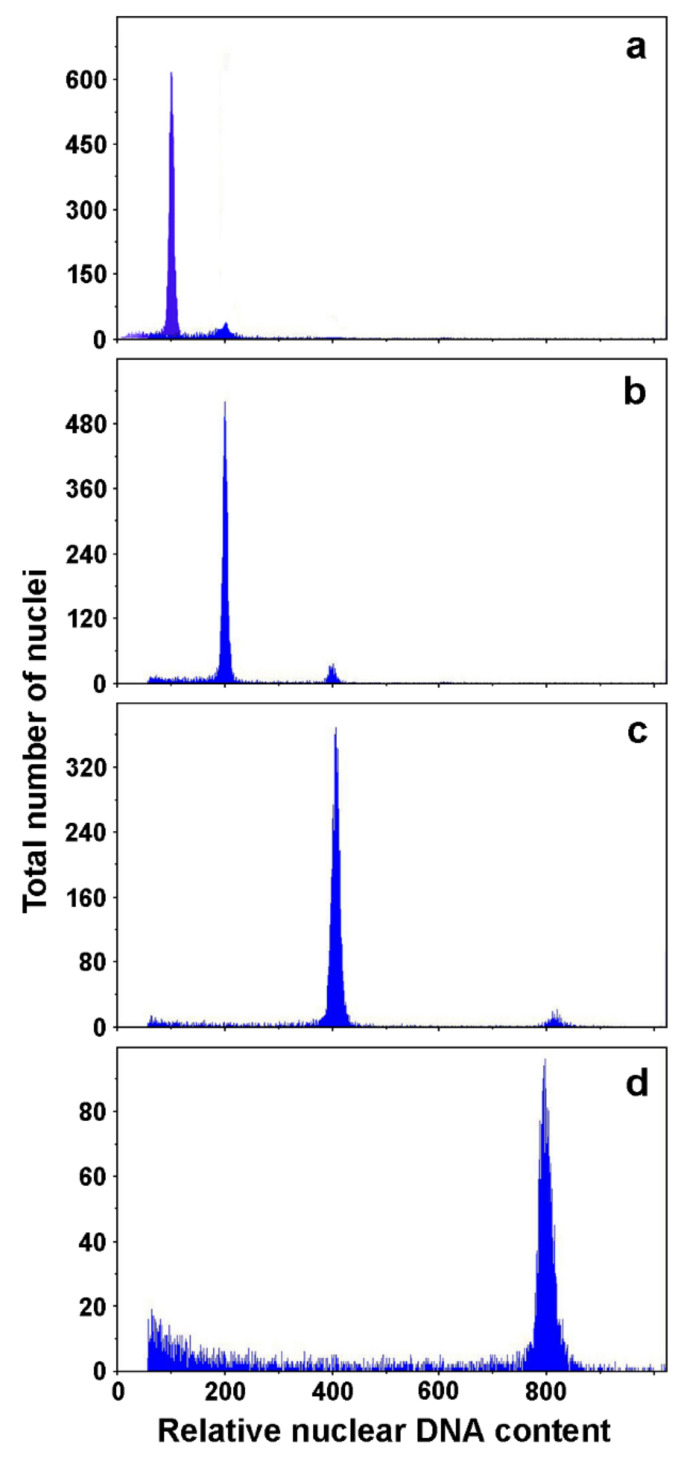
Flow cytometry analysis of microspore suspension-derived androgenic embryos of *A. hippocastanum*: (**a**) haploid; (**b**) diploid; (**c**) tetraploid; (**d**) octaploid. Adapted from Ćalić-Dragosavac et al. [106].

Ploidy level of anther culture- and microspore suspension-derived embryos was determined under cytological examination [23,97,98,100,101] or by flow cytometry [99,105]. In *A. carnea* anther culture, 43% of tested plantlets were haploids, while the rest were diploids or in rare cases aneuploids [101]. Similarly, in *A. hippocastanum* 50% of tested regenerants obtained from anther culture were haploids, while 100% of regenerants derived from microspore suspensions were haploid [98]. In a later study it was shown that the first generation of microspore-derived androgenic embryos were haploid in 100% tested cases [106]. However, after 3 years of maintenance by secondary somatic embryogenesis only 10% of the tested embryos were haploid, whereas 10.5% were diploid, 73.5% tetraploid and 6% octaploid [106] (Figure 3). Among the anther-culture derived embryos, after 3 years of cultivation there were no haploids, whereas 8.5% were diploid, 81% tetraploid and 10.5% octaploid.

Except for the origin and chromosome number, AEs do not differ from SEs in respect to further development and multiplication (Figure 1 and Figure 2). AEs can multiply by forming secondary embryos through direct embryogenesis. Because they originate from somatic cells of the primary AE, the newly formed secondary embryos are considered SEs despite the fact that they have a haploid number of chromosomes [95].

A rather high level of albinism (Figure 2f), ranging from 11–14% [105] to even 21% [100], is associated with embryo regeneration from anther and microspore suspension culture in *Aesculus* sp. [100]. It has recently been shown that albinism appeared as a consequence of transcriptional repression of the key genes required for proper chloroplast biogenesis during the first 7–21 days of in vitro cultivation [107]. In horse chestnut, abscisic acid (ABA) at low levels (0.01 mg L^−1^) decreased the frequency of albino embryos from 11% to 6% and chimeric embryos from 6% to 2% in microspore suspension culture and albino embryos from 8% to 4% in anther culture [105]. In albino embryos, nuclei were degraded, which caused cessation of embryo growth. By contrast, nuclei of green embryos were clearly stained with 1% 4, 6-diamidino-2-phenylindole (DAPI) [105].

Since SEs and AEs share the same course of embryo multiplication and further development, their secondary somatic embryogenesis, embryo maturation, germination and conversion to plantlets will be described concurrently.

### 3.3. Secondary Somatic Embryogenesis

Zygotic, somatic, androgenic and gynogenic embryos are all capable of a new round of embryo regeneration. Since the newly formed embryos regenerate from somatic cells, they are called somatic embryos, regardless of the origin of the primary embryo, and the process is called repetitive, recurrent, adventive or secondary somatic embryogenesis [108]. Secondary somatic embryogenesis was reported from the very beginning of the research of somatic embryogenesis [76,79,80] and androgenesis [97] in *Aesculus* sp. This is a very powerful means of embryo amplification, as one SE could regenerate dozens of secondary somatic embryos (SSEs), thus increasing the number of SSEs exponentially with each round of secondary somatic embryogenesis. For instance, up to 45 SSEs were recorded per primary somatic embryo (PSE) after 4 weeks of culture in *A. flava* [86], and 20–30 SSEs per PSE for five weeks of culture in *A. hippocastanum* [77]. As soon as SEs regenerate from embryogenic calli, they start regenerating SSEs, so it is not possible to distinguish PSEs from SSEs (Figure 1g and Figure 2e) [86]. SSEs regenerate from the subepidermal layer of PSEs [109], mainly from the base and the tip of a radicle by direct somatic embryogenesis [86,109].

AEs and SEs of *Aesculus* sp. spontaneously regenerated SSEs on medium devoid of PGRs [86,87,109,110], but the frequency of secondary somatic embryogenesis could be rather low, as was observed in *A. carnea* (3–39%) [87]. Cytokinins (1–10 μM BA or Kin) significantly amplified this process, increasing PSE response to 81% in *A. carnea* [87]. Similarly, Jörgensen [76] applied 2.5–5 μM BA for SSE induction in *A. hippocastanum*, whereas Kiss et al. [77] used a rather high concentration of BA (44 μM) for this purpose, but which required subculture of PSEs onto a medium containing lower levels of BA (2.2–8.8 μM) 4–5 days later to avoid SSE malformations.

In *A. hippocastanum*, PGR-free medium was superior for SSE formation from AEs compared to media supplemented with different combinations of activated charcoal (AC), ABA, polyethylene glycol (PEG) and mannitol [110]. However, SSEs derived from horse chestnut AEs cultivated on PGR-free medium had rather high levels of morphological abnormalities [109]. ABA at 0.01 mg L^−1^ enabled the production of better-quality embryos, although at the expense of SSE number, causing a three-fold decrease in SSE number compared to PGR-free medium. Higher levels of ABA (0.1–5 mg L^−1^) caused a significant, eight-fold decrement of SSE number compared to 0.01 mg L^−1^ ABA [109].

The ability of PSEs to form SSEs was most significantly impacted by the stage of PSE development [77,86,87,109]. PSEs at the globular and heart-shaped stage of development exhibited the highest capacity for SSE initiation, reaching 99% in *A. hippocastanum* [109], and 74% in *A. flava* [86] and *A. carnea* [87]. As the PSEs grew, they gradually lost the ability for SSE formation [77,87]. However, 10 mm-long PSEs of *A. carnea* were capable of both SSE and adventive bud regeneration, while germinated PSEs (30 mm) almost exclusively formed buds [87]. The processes of adventive somatic embryogenesis and shoot organogenesis from the same PSE seldomly occurred in parallel (only 2.5–12.8% of PSEs, depending on the stage of development) [87]. BA and Kin had a similar effect on SSE induction, whereas BA promoted shoot organogenesis more efficiently than Kin. The root pole of PSEs exhibited the highest embryogenic response [87].

### 3.4. Maturation and Germination of Embryos and Their Conversion to Plants

Although great success has been achieved in the induction of androgenesis and somatic embryogenesis in a few *Aesculus* species, further development of embryos, including germination and conversion into plantlets, was generally poor with all protocols and species, causing a bottleneck in the regeneration of whole plants. In the first reports, encouraging results on the conversion of SEs into plants were presented. Gastaldo et al. [82] reported on the formation of whole plantlets from SEs induced from horse chestnut stem explants. These SEs were not dormant and immediately produced plantlets [82]. The SEs first developed a long root, then plumules, just like during seed germination in nature. However, when SEs were induced from cotyledons isolated from zygotic embryos of ripe seeds, they were dormant and required chilling at 6 °C in darkness for 6 months to break the dormancy [81]. Upon their return to room temperature, 100% of the SEs germinated and then developed plumules, while SEs cultivated constantly at 25 °C formed neither roots nor shoots [81]. The authors assumed that SEs obtained from the cotyledons of dormant seeds are dormant themselves, and found that cotyledons were responsible for the dormancy, since the embryonic axes (embryos without cotyledons) of *A. hippocastanum* isolated from mature seeds and cultured in vitro were not dormant and developed into plantlets [111]. However, further development, acclimatization and establishment of the plants in soil were not reported in these studies.

Later it became clear that conversion of SEs into plantlets is quite a challenge in *Aesculus* species. In *A. glabra* and *A. x arnoldiana* radicle elongation of SEs was observed, but plumules seldomly developed [85]. By contrast, SEs induced from the first true leaves of *A. hippocastanum* seedlings developed plumules at the frequency of approximately 80%, but the root system rarely developed and was very poor [80]. In *A. glabra* approximately 10% of the embryos germinated, forming both roots and shoots, but the shoots did not produce more than a few leaves [84]. Similarly, in filament-derived SEs of *A. hippocastanum* only up to 1% converted to plantlets [77].

**Figure 4:**
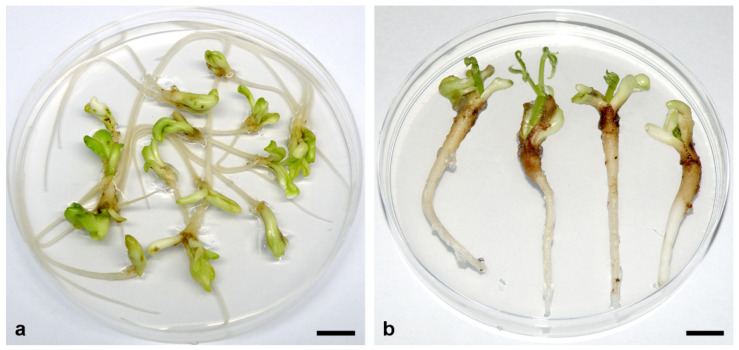
Somatic embryo germination in *A. flava*. (**a**) Somatic embryos at the cotyledonary stage of development (~1 cm long) germinated and developed long primary roots after 4 weeks of cultivation on MS medium supplemented with 0.05 μM 2,4-D + 5 μM Kin. (**b**) Plumule development after an additional month of cultivation on the same medium. Scale bars = 1 cm. Unpublished from the authors’ Laboratory.

In *A. flava*, more than 80% of 7–10 mm SEs cultivated on MS medium supplemented with 0.05 μM 2,4-D + 5 μM Kin or BA developed roots (Figure 4a), and up to 65% of these SEs also developed epicotyls without chilling treatment (Figure 4b) [86]. Although the frequencies of root formation and conversion to plantlets did not differ significantly between SEs cultivated on medium supplemented with BA or Kin, SEs cultivated on Kin-supplemented medium showed longer roots and shoots [86]. However, further development of all plantlets was arrested, as was observed in *A. glabra* [84].

Embryo maturation is the key phase, as it affects embryo germination and subsequent plant development. It is during this stage that SEs undergo significant changes which affect correct deposition of storage materials, the repression of precocious germination and acquisition of desiccation tolerance [108]. Consequently, desiccation treatments were included in subsequent studies in order to improve the maturation and post-maturation phases [78,88]. Testing the effects of AC, ABA, PEG and mannitol at several concentrations, either individually or in combination, Capuana and Debergh [88] recorded the highest number of mature cotyledonary SEs when embryogenic calli were cultivated on medium supplemented with 10 μM ABA alone or with 0.1% AC + 5% PEG-4000. These treatments yielded the highest proportions of morphologically normal SEs (72% and 70%, respectively). The same number of embryogenic calli cultivated on medium without adjuvants produced approximately 2.5-fold fewer cotyledonary SEs, only half of which were morphologically normal. Slow desiccation (in empty, non-sealed Petri dishes in laminar air flow for 24–48 h) further improved SE development, but germination (15%), shoot elongation (13.7%) and conversion (4.8%) rates were still generally low [88]. The highest conversion rate obtained in the study was 10%, using 80 μM ABA with slow desiccation.

Troch et al. [112] showed that the vascular system and shoot and root apices in horse chestnut SEs were well-established under histological examination. Hence, they concluded that factors other than improper shoot meristem organization were responsible for poor germination and conversion. By varying both sugar type and concentration, Troch et al. [112] achieved high conversion rates and obtained high percentages of embryos forming both a shoot and a root (up to 88.9%). However, according to the authors, the overall quality of the plantlets was rather poor, and the subsequent growth and acclimatization of these plants was not reported. The inclusion of PEG in maturation medium decreased the conversion percentages, but improved the quality of the shoots, particularly in combination with sucrose. Cold treatment was also useful for overcoming embryo dormancy [81,112,113].

For efficient germination, anther- and microspore-culture-derived embryos of horse chestnut required 6 months of chilling at 6 °C [113]. Despite a rather high germination frequency (76.6%), only a small proportion of these embryos (2–9.6%) converted to plantlets [99,113]. Since chilling itself (30–90 days) was not sufficient for efficient germination and conversion of embryos to plantlets, AC, ABA and PEG, singly or in combination, were used to improve the germination and conversion rates. The horse chestnut AEs cultivated on medium supplemented with 1% AC developed roots at the frequency of 99%, while 18% and 12% of anther- and microspore suspension-derived embryos, respectively, converted to plantlets [106]. ABA, PEG, ABA+AC and PEG+AC were not effective for embryo conversion, reaching a maximum of 7%.

Attempts to establish somatic seedlings of *Aesculus* sp. in the soil for longer periods of time has not been reported to date. Despite numerous reports on acclimatization of the somatic seedlings, it seems that these plants survived in the soil only briefly, as was reported for *A. glabra* [84] and *A. flava* [86]. The plantlets died mainly due to fungal infections despite fungicidal treatment [86]. To the best of our knowledge, there are no reports on mass acclimatization of somatic seedlings for any *Aesculus* species. However, *Aesculus* species are not the only example of unsuccessful acclimatization of somatic seedlings. The establishment of in vitro-derived plants in the soil is very difficult in numerous hardwood species [114,115]. However, successful acclimatization and good performance in the field trials of certain hardwood species, such as *Querqus suber* [25], are encouraging for further attempts to overcome this problem in *Aesculus* sp.

### 3.5. Cryopreservation of Somatic Embryos

Embryogenic cultures usually lose their embryogenic capacity over time, and the level of somaclonal variations increases with prolonged cultivation [108]. Thus, cryopreservation of newly established embryogenic cell lines is highly recommended [69,73,108]. Among *Aesculus* species, cryopreservation was successfully applied only to embryogenic cultures of *A. hippocastanum* [116,117,118]. In early studies a two-step cooling procedure for cryopreservation of embryogenic cultures was used [116,117], with an efficient one-step freezing procedure established afterwards [118]. At first, only SEs at the globular stage of development were subjected to cryopreservation [116,117], but later it was shown that proliferating embryogenic calli, consisting of embryogenic masses at the pre-globular stage and SEs at different stages of development, were much better material for cryopreservation [118].

For preconditioning to enhance chilling and desiccation tolerance, Jörgensen [116] used a simple cryoprotectant mixture containing 0.51 M DMSO + 0.15 M sucrose, while Jekkel et al. [117] used a mixture of 0.5 M DMSO + 1 M sucrose + 0.5 M glycerol. In both studies a similar cooling procedure was used, in which globular SEs were incubated in a cryoprotectant solution at 0 °C for 1h, then the cultures were cooled to −35 °C to −40 °C at the rate of 0.5–1 °C min^−1^, and finally submerged into liquid nitrogen. This procedure enabled a high recovery rate of approximately 90% of SEs, but these SEs were not able to develop further or to form SSEs [116]. However, Jekkel et al. [117] found ABA pretreatment for 4 days before cryopreservation to be crucial for successful SE recovery, since only SEs pretreated with different ABA concentrations (0.75–75 μM) survived the cryopreservation. Among all ABA treatments, SEs pretreated with 0.75 μM ABA exhibited the highest recovery rate of 43%. Thawed SEs not only recovered growth ability, but 3% of them also multiplied by secondary somatic embryogenesis. Jekkel et al. [117] first attempted a fast-freezing procedure with air-dried horse chestnut globular SEs, which were directly immersed in liquid nitrogen without a cryoprotectant. SEs subjected to open air flow under aseptic condition lost a significant amount of water, with moisture contents of 53%, 22% and 13% after 2, 3 and 4 h, respectively, of desiccation in a laminar hood. The highest recovery rate (46%) was attained in SEs dried for 4 h and pretreated with 0.75 μM ABA.

Further progress was made by using proliferating embryogenic calli with SEs at different stages of development [118]. In vitrification/one-step procedure, the embryogenic cell masses were preincubated on proliferation medium at 4 °C in the dark for 5 days, dehydrated in solution containing 2 M glycerol + 0.4 M sucrose at 25 °C for 30 min and then in Plant Vitrification Solution No. 2 (PSV2) containing MS medium with 30% glycerol + 15% ethylene glycol + 15% DMSO + 0.4 M sucrose at 0 °C for 60 min, and finally immediately frozen in liquid nitrogen. After thawing at 40 °C, the frequency of tissue recovery ranged from 32.5%, for embryogenic calli with globular SEs, to 75% for embryogenic calli with torpedo SEs. The frequencies of recovery of embryogenic calli with torpedo SEs was further increased to 93.3% by prolonging incubation of the tissue in PSV2 for 90 min or to 94.1% by increasing the thawing temperature to 45 °C.

### 3.6. De Novo Shoot Organogenesis

Induction of de novo shoot organogenesis has been rarely practiced in *Aesculus* species, although it proved to be rather efficient in several studies. High shoot regeneration frequencies were attained from stem discs of in vitro-cultivated shoots in *A. hippocastanum* [119], from shoot tips excised from shoots harvested from a 15-year-old tree [120] and a 40-year-old tree [121] of *A. carnea*, and from germinated SEs (i.e., somatic seedlings) of *A. carnea* [87] and *A. hippocastanum* [122] (Table 3). Cytokinins were sufficient for the induction of caulogenic response, with BA being the most efficient in the majority of studies [87,119]. Different explants had various PGR requirements and exhibited different caulogenic responses. Somatic seedlings and stem segments of in vitro-cultivated plantlets showed the highest caulogenic response [87,119]. A summary of the literature data on de novo shoot induction in *Aesculus* species is given in Table 3.

#### 3.6.1. Induction of Shoot Regeneration

In *A. hippocastanum*, BA and TDZ induced direct adventive shoot regeneration from leaf blades, petioles and stem discs of in vitro-cultivated shoots [119]. BA was more efficient than TDZ in all explant types. The highest response was attained from stem discs (up to 100%), and the lowest from the leaf blades (up to 46.6%). For stem discs and leaf blades, the optimal TDZ concentration was 1.1 μM, while petioles required a higher level of TDZ (2.2 μM). BA at 4.4 μM provoked response in 100% of stem discs and resulted in the highest number of shoots (8.3 on average) [119]. For shoot induction from leaf blades, higher levels of BA (4.4–8.9 μM) were required, while 2.2 μM BA was optimal for shoot induction from petioles. The addition of 0.5 μM NAA to BA-supplemented media did not improve regeneration response of the three explant types, with the exception of stem discs, in which 100% response and the highest number of shoots (9.7) was attained from explants cultivated on medium supplemented with 8.9 μM BA + 0.5 μM NAA for four weeks [119].

In *A. carnea*, BA was also used for de novo shoot organogenesis from shoot tips isolated from adult trees [120,121]. For shoot induction, Masubuchi [120] used 5 μM BA + 0.1 μM indole-3-butyric acid (IBA), while Evtushenko et al. [121] applied 2.2 μM BA + 2.3 μM Kin or 2.2 μM BA + 2.5 μM IBA and obtained 32.2 and 26.8 shoots per explant, respectively, in six weeks.

**Figure 5:**
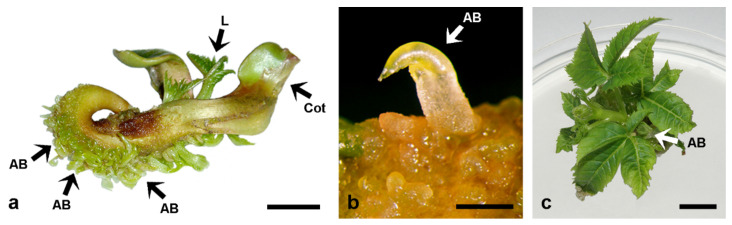
De novo shoot bud induction and plant regeneration in *A. carnea*. (**a**) Numerous adventitious shoot buds regenerated from the hypocotyl of somatic seedlings (~3 cm) cultivated on MS medium supplemented with 5 μM BA: AB, adventitious bud; Cot, cotyledon; L, primary leaves. (**b**) Detail of **a**, showing an adventitious bud (AB) emerging directly from the hypocotyl of the seedling. (**c**) Plant developed from a single adventitious bud after 6-week cultivation on MS medium supplemented with 5 μM BA. Adventitious buds (AB) regenerated from the shoot base. Scale bars: 1 cm (**a**,**c**); 1 mm (**b**). Unpublished from the authors’ Laboratory.

Somatic seedlings of *A. carnea* longer than 10 mm, cultivated on MS media supplemented with 1, 5 or 10 μM BA or Kin, regenerated adventitious buds predominantly along the hypocotyls (Figure 5a) and only occasionally on the cotyledons and roots [87]. The shoots regenerated directly from the seedling tissues (Figure 5b) and were able to develop further when cultivated on medium supplemented with 5 μM BA or Kin (Figure 5c). These shoots also developed secondary shoots at the base of their stems (Figure 5c). BA always induced a higher caulogenic response in explants than Kin. The stage of somatic seedling development was highly important for shoot induction. The highest caulogenic response was obtained from 30 mm-long somatic seedlings cultivated on MS medium supplemented with 10 μM BA, which responded with the frequency of 99.1% and 68.8 buds per explant, while explants cultivated on MS medium with 10 μM Kin regenerated shoots at significantly lower frequency (65.8%) and mean bud number per explant (5.0) [87].

BA also efficiently promoted shoot formation from somatic seedlings of *A. hippocastanum*. BA at concentrations of 5 and 10 μM induced adventive bud formation in 95% and 100% of somatic seedlings, with 12.3 and 20.27 adventitious buds per seedling, respectively [122]. However, 10 μM BA frequently caused hyperhydration of the adventive shoots, thus 5 μM BA was recognized as a better choice for efficient induction of healthy shoots. Adventitious shoots regenerated from the seedlings, referred to as the primary shoots, developed numerous secondary shoots in the presence of 0–20 μM BA over the course of four weeks [122]. Again, 10 μM BA was the most efficient for secondary shoot induction, but also caused hyperhydration of secondary shoots. A high incidence of hyperhydricity was also reported in stem disc-derived horse chestnut shoots cultivated on media supplemented with TDZ or BA at concentrations higher than 1.1 μM [119]. BA quite often caused hyperhydricity in numerous plant species [123].

#### 3.6.2. Shoot Elongation and Rooting, and Physiological Disorders in Regenerated Plants

The most frequent physiological disorders seen in the regenerated plantlets were hyperhydricity and necrosis of the shoot apex. Shoot-tip necrosis (browning of the apical shoot) is a rather complex phenomenon, most probably caused by the synergistic action of multiple factors, including calcium and boron deficiencies, and suboptimal levels of cytokinins in the shoot apex or their decreased availability due to the conversion of active cytokinin forms to inactive and toxic 9-glucosides [124,125,126]. Therefore it is rather difficult to find a good balance between achieving efficient shoot production and elongation and minimizing the level of the accompanying disorders. In *A. hippocastanum* shoots cultivated on MS medium supplemented with 0–2.5 μM BA, necrosis of the shoot apex occurred at a very high frequency (100% and 76%, respectively), thus the shoots required BA at higher levels than 2.5 μM, which in turn suppressed shoot elongation and rooting [122]. Hence, individual shoots of *A. hippocastanum* were elongated on solid MS medium with 1 μM BA and 500 mg L^−1^ polyvinylpyrrolidone (PVP, MW 40,000) for 4 weeks [122]. Individual shoots elongated at a frequency of 63% [122]. For root induction, the basal part of elongated shoots was wounded by cutting with a sterile blade, dipped into a 0, 5 or 10 mM IBA solution for 1 min, and placed on half-strength MS, PGR-free solid medium supplemented with 0.02% AC for 2–3 weeks [122]. IBA was necessary for root induction, as spontaneous rooting was not observed. The highest rooting rate (23%) was observed in shoots treated with 10 mM IBA, whereas in those treated with 5 mM IBA it was only 8.3%. The rooted shoots had well-developed root systems, with a 10–12 cm-long main root and numerous lateral roots. To prevent shoot-tip necrosis during the rooting phase, 10 μL of 0, 1, 5 or 10 μM BA solution was applied directly to the apical meristem of the shoots. As soon as the root initials were observed, the shoots were transferred to MS medium supplemented with 500 mg L^−1^ PVP + 5 μM BA. The application of 1 μM BA on the apical meristem weekly during the root induction phase was the optimal treatment. Higher levels of BA or higher frequency of BA application caused hypertrophy of the shoot tip, while a prolonged interval of BA application did not prevent necrosis of the shoot apex [122].

Šedivá et al. [119] used 1.04 μM meta-Topolin for elongation of stem discs-derived horse chestnut shoots and did not observe shoot-tip necrosis during the elongation and rooting phase. In their study, NAA was more effective than IAA in root induction, with the highest rooting rate of 68% and the mean root number per explant (4.2) attained in the shoots cultivated on WPM supplemented with 2.7 μM NAA. However, the highest survival rate of the plants in a greenhouse (100%) were from plantlets rooted on WPM supplemented with 4.8 μM IAA. To the best of our knowledge this is the most successful rooting procedure in any *Aesculus* species to date, resulting in healthy-looking plants with the longest reported survival time of the plants in the soil. This success could be due to the use of meta-Topolin, given that topolins are as effective, but less harmful, than BA [127,128] (which adversely affects rooting and subsequent acclimatization of micropropagated plants in some species [129]).

A rather high rooting rate of 53% was achieved in the microshoots of *A. carnea* cultivated on 1/4 WPM supplemented with 0.1 μM IBA + 10 g L^−1^ AC. However, acclimatization success of these plants was not reported [120].

## 4. Hairy Root Cultures of *Aesculus* sp.

To date, *A. glabra* [84] and *A. hippocastanum* [130,131] are the only members of the genus *Aesculus* that have been genetically transformed. However, hairy roots were obtained only in *A. hippocastanum*. Hairy roots are often used for the production of secondary metabolites [132,133,134] because of their immensely high biomass production, the capacity for long-term growth [130] and both genetic and biochemical stability [132].

Horse chestnut hairy root lines were obtained by *Agrobacterium rhizogenes*-mediated genetic transformation of somatic embryos of *A. hippocastanum* at the cotyledonary stage of development [130,131]. To achieve this, strain A4GUS of *A. rhizogenes*, harboring the pRiA4 non-disarmed plasmid with the *uidA* gene construct integrated into the TL-DNA between the *rolC* and *rolD* genes [135], was used. Hairy roots emerged from the wound sites, mostly at the basal parts of cotyledons, within a month of inoculation. The presence of 50 µM acetosyringone during co-cultivation of the explants with bacteria doubled the number of putative transformants (10.0% vs. 5.3% in its absence) [131]. Seventy-one hairy root lines were obtained from independent transformation events, but only five lines exhibited long-term sustained growth [130,131], being maintained for more than 20 years. The hairy root lines exhibited a transformed phenotype: vigorous growth in PGR-free medium, high branching and the loss of the gravity response, while non-transformed roots of *A. hippocastanum* perished after two to three subcultures on PGR-free medium [130]. The mass of the five hairy root lines increased six- to eight-fold over the initial mass within a four-week culture period [130]. PCR analysis confirmed the presence of the *rolA*, *rolB*, *rolC* and *rolD* genes in all five well-growing lines, assuring their vigorous growth [130]. Finally, Southern blot hybridization confirmed the stable integration of the TL-DNA and the integration of two to four copies of the TL-DNA in the horse chestnut genome [131].

## 5. Aescin Production from In Vitro Cultures

Given that the cotyledons of the zygotic embryos of *Aesculus* sp. are the main site of aescin accumulation within the plant, they were expected to produce a similar array of compounds, including aescin, under in vitro conditions. Cotyledonary fragments isolated from ripe horse chestnut seeds formed calli even in the absence of PGRs. These calli still contained aescin after 17 weeks of cultivation [136]. The addition of 2,4-D, NAA or GA_3_ (each at 0.5 mg L^−1^) in the culture medium doubled the level of aescin in cotyledon-derived calli, although there were no significant differences among the treatments [136]. In later studies, a significant amount of aescin (approximately the same level as in ripe cotyledons) was found in non-embryogenic calli induced from the primary leaves on MS medium supplemented with 9.3 μM Kin + 10.7 μM NAA + 9 μM 2,4-D, while its level was three- to five-fold higher in embryogenic calli and embryos [137]. Actually, the origin of calli and somatic embryos was not important for aescin production in vitro, since calli and SEs obtained from cotyledons, stem explants or primary leaf fragments produced aescin at similar levels [138]. Most importantly, SEs contained aescin at levels similar to those of cotyledons extracted from the ripe seeds of horse chestnut [138]. It was concluded that in vitro-cultured horse chestnut tissue would be an excellent source of aescin, since it contained similar or even higher levels of aescin than horse chestnut seeds [138]. However, this material has never been exploited for industrial production of aescin, despite the estimation that the extraction of aescin from bioreactor-cultivated tissue would be easier and less expensive than from natural sources [138]. A substantial amount of aescin was also found in horse chestnut AEs at the cotyledonary stage of development [139]. AEs at the cotyledonary stage of development cultivated on PGR-free medium were 2.43% aescin by dry weight, while the level of aescin was very low in AEs at the globular stage of development (0.59%) [139]. However, AEs at the cotyledonary stage of development cultivated on MS medium supplemented with 2,4-D, NAA or ABA (each at 0.5 mg L^−1^) contained 6.77%, 6.03% and 6.0% aescin, respectively [139]. In this study, ripe zygotic embryos contained 6.96% aescin. Other PGRs, such as IBA, Kin and BA (each at 0.5 mg L^−1^) also increased the level of aescin in AEs (4.66–5.45%). The level of aescin increased with increased concentration of these PGRs (0.1–0.5 mg L^−1^), probably in response to stress [140].

As aescin was also detected in native roots of *A. assamica* [34], *A. turbinata* [141] and seedling roots of *A. parviflora* [142], horse chestnut hairy roots were also tested for the presence of aescin. Four best-growing horse chestnut hairy root lines cultivated in liquid PGR-free medium produced aescin in variable amounts (1.16–4.09%), with the highest content detected in the two lines which were also the best biomass producers [139]. Lower aescin productivity of hairy roots, compared to aescin levels found in zygotic embryos (6.96%), might be compensated for by the high biomass production of hairy roots. Furthermore, due to the expression of the *rol* genes, hairy root cultures do not require any PGRs, which are the most expensive culture medium ingredients; they can therefore be easily maintained in a liquid medium, thus making culture maintenance highly economical.

Besides aescin, noticeable amounts of esculin and esculetin were detected in embryogenic calli and SEs obtained from horse chestnut bark explants [83]. Esculin and esculetin are coumarins, which are also used for the treatment of blood vessels disorders. The concentration of esculin in SEs was 30–40% lower than in bark tissue isolated from horse chestnut trees, while the level of esculetin in SEs was very similar to that of the bark control [83].

## 6. Conclusions and Prospects

We presented here a short survey of aescin content in *Aesculus* species and its medical uses, as well as a comprehensive review of in vitro propagation methods established in these species. Somatic embryogenesis, androgenesis and de novo shoot organogenesis have been successfully achieved in several *Aesculus* species. The obtained protocols represent a good base for application of these methods for the induction of in vitro regeneration in other *Aesculus* species, cultivars and hybrids. Elite specimens selected as the best aescin producers, elite ornamentals or specimens resistant to *C. ohridella* infestation could be easily cloned and propagated by these techniques. Furthermore, the developed methods are suitable for genetic transformation and molecular breeding of these species. In view of this, somatic embryos are ideal targets for genetic transformation, while secondary somatic embryogenesis is an ideal process for whole plant recovery from the genetically transformed cells.

Androgenesis is also efficiently and routinely used for haploid embryo induction, allowing for subsequent dihaploid production. As was shown, dihaploids arose spontaneously from microspore suspension culture, without the requirement for induced diploidization. Dihaploids, as pure lines, are particularly important for breeding purposes, especially in woody plant species.

In addition, secondary somatic embryogenesis and adventive shoot organogenesis enable mass production of embryos and shoots, with a high potential for further use in planting material and aescin production. *Aesculus* species have been propagated mainly by seeds and stem cuttings with limited success. More importantly, the seeds are not suitable for clonal propagation of elite specimens, since they are mainly sired by cross-pollination. Thus, the abovementioned tissue culture techniques are a very promising alternative for the production of planting material. However, poor germination, conversion and acclimatization rates, and difficulties establishing acclimatized plants in the soil are currently huge constrains and an obstacle to sustained production of the planting material. Despite this, significant improvements have been achieved in the maturation phase, enabling regeneration of morphologically normal SEs at a high frequency (70%), along with a rather high embryo germination frequency (80% or higher), and even higher conversion rates (nearly 90%). Unfortunately, the overall quality of the obtained plantlets was still poor, and their further development was arrested. Attempts to establish somatic seedlings of *Aesculus* sp. in the soil for longer periods of time failed, thus their mass production in the field has never been achieved. Hence, further efforts to optimize these processes in *Aesculus* sp. are required.

A substantial amount of aescin detected in somatic embryos (comparable to the amount found in the zygotic embryos—the main site of aescin accumulation within the plant) is of great importance. This opportunity has not been exploited yet for the industrial production of aescin. Currently, aescin is produced on an industrial scale from horse chestnut seeds, with a few patents proposed for *A. chinensis* and *A. indica*. A horse chestnut tree can produce up to 1600 seeds [65], i.e., the same number of cotyledonary zygotic embryos. In our laboratory, up to 3000 SEs can be produced starting from 100 mg of embryogenic cell aggregates in a suspension culture of *A. flava* in three weeks (data not published yet), and the optimization of the procedure for cotyledonary somatic embryo production is in progress in our laboratory.

Furthermore, tissue culture-derived plant material is available all year round, and a successful cryostorage procedure assures constant availability of plant material. In addition, chemical analyses have shown the ubiquitous presence of aescin in all *Aesculus* species, and their traditional uses by local populations give evidence of their ability to cure numerous medical disorders. However, some of these species have a rather small areal, thus the number of nuts they produce might be insufficient for industrial use. Every *Aesculus* species produces a specific mixture of aescins; the exclusive use of horse chestnut seeds commercially means only a small percentage of aescins are being exploited. In vitro cultivation of tissues of other *Aesculus* species offers an array of new compounds, which would lay a very good foundation for the production of new drugs.

As demonstrated above, PGRs significantly and positively affected aescin production in SEs, indicating that aescin production might be manipulated and further increased by the use of elicitors. Finally, the presence of heavy metals, mycotoxins, pesticide residues, polycyclic aromatic hydrocarbons, fumigants and other contaminants in herbal preparations has been documented [143,144]; phytochemical production from tissue culture-derived plant material comes with no such risks and could be considered a clean and safe technology.

We hope that this paper will put these opportunities in the focus of other researchers, with the aim of better exploitation of the natural resources of *Aesculus* species.

## Figures and Tables

**Table 1:** Summary of the literature data on indirect somatic embryogenesis initiation from various explant types isolated from different sources in *Aesculus* species. Plant growth regulator content of media used for embryogenic callus induction and embryo regeneration is given in the table.

Species/ Hybrid	Explant Source	Explant Type	Callus Induction	Culture Conditions * (Light/Dark, Temperature)	SE Regeneration	Reference
*A x arnoldiana*	4-week-old in vitro seedlings	shoot	WPM + 25 μM BA	Dark, 25 °C	WPM + PGR-free	[85]
	4-week-old in vitro seedlings	root	WPM + 5 μM BA	Dark, 25 °C	WPM + PGR-free	[85]
	30-year-old tree	shoot	WPM + 25 μM BA	Light, 25 °C	WPM + PGR-free	[85]
*A. glabra*	3-year-old trees	shoot	WPM + 5 μM BA	Dark, 5 °C	WPM + PGR-free	[85]
	3-week-old in vitro seedlings	stem, petiole, leaf blade	MS + 4.5 μM 2,4-D + 4.7 μM Kin	Light, n.s.	MS + PGR-free	[84]
*A. flava*	flower buds	filaments	MS + 1 μM 2,4-D + 10 μM Kin	Dark, 25 °C	MS + PGR-free	[86]
*A. hippocastanum*	seedlings	primary leaves	MS + 9.3 μM Kin + 10.7 μM NAA + 9 μM 2,4-D	Light, 25 °C	MS + PGR-free	[80]
	young seeds	immature zygotic embryos	MS + 13.6 μM 2,4-D + 4.6 μM Kin	n.s.	MS + 4.5 μM 2,4-D + 4.6 μM Kin	[79]
	flower buds	filaments	WPM + 2.5 μM BA + 5 μM 2,4-D	Light, 25 °C	WPM + PGR-free	[76]
	zygotic embryos of ripe seeds	cotyledons	MS + 0.45 μM 2,4-D	Light, 25 °C	MS + PGR-free	[81]
	immature zygotic embryos, filaments	somatic embryos	MS + 8.8 μM 2,4-D + 5.4 μM NAA	Dark, 28 °C	B5 + 4.4 μM BA	[77]
	terminal branches of an adult tree	stem segments	MS + 9.3 μM Kin + 10.7 μM NAA + 9 μM 2,4-D	Light, 25 °C	MS + PGR-free	[82]
	terminal branches of an adult tree	bark fragments	MS + 9.3 μM Kin + 10.7 μM NAA + 9 μM 2,4-D	Light, 25 °C	MS + PGR-free	[83]
	flower buds	filaments	MS + 9 μM 2,4-D	Dark, 23 °C	MS + PGR-free	[78,88]

SE—somatic embryo; PGR—plant growth regulator; BA—benzyladenine; 2,4-D—2,4-dichlorophenoxyacetic acid; Kin—kinetin; NAA—α-naphthaleneacetic acid; MS—Murashige and Skoog mineral solution; WPM—woody plant medium; B5—Gamborg mineral solution; n.s.—not specified. * Culture conditions refers to callus induction phase.

**Table 2:** Summary of the literature data on androgenesis induction in *Aesculus* species. The table shows plant growth regulators used for embryogenic callus induction and androgenic embryo regeneration, as well as the method used for determination of ploidy level in regenerated plantlets.

Species/ Hybrid	Flower Bud Size (mm)	Regeneration Method	Callus Induction	Culture Conditions *	Embryo Regeneration	Ploidy Level Determination	Reference
*A. carnea*	4–6	ANC	MS + 4.5 μM 2,4-D + 4.6 μM Kin	Light, 25 °C	MS + 0.045 μM 2,4-D + 4.6 μM Kin	Chr.count.	[100]
	4–7	ANC	MS + 0, 0.45, 6.8 or 9.1 μM 2,4-D + 4.6 μM Kin	Light, 25 °C	MS + 0.045 μM 2,4-D + 0.46 μM Kin	Chr.count.	[101]
*A. flava*	4–12	ANC	MS + 4.5 μM 2,4-D + 4.6 μM Kin	Dark, 25 °C	MS + 0.045 μM 2,4-D + 4.6 μM Kin	n.t.	[102]
	4–5	ANC and MSC	MS + 4.5 μM 2,4-D + 4.6 μM Kin	Dark, 25 °C	MS + 0.045 μM 2,4-D + 4.6 μM Kin	n.t.	[103]
*A. hippocastanum*	4–7	AC	MS + 4.5 μM 2,4-D + 4.6 μM Kin	Light, 28 °C	MS + PGR-free	Chr.count.	[97]
	4	ANC and MSC	MS + 4.5 μM 2,4-D + 4.6 μM Kin	Dark, 25 °C	MS + 0.045 μM 2,4-D + 4.6 μM Kin	Chr.count.	[98]
	4	ANC and MSC	MS + 4.5 μM 2,4-D + 4.6 μM Kin	Dark, 23 °C	MS + 0.045 μM 2,4-D + 4.6 μM Kin	Flow cyt.	[99]

ANC—anther culture; MSC—microspore suspensions; MS—Murashige and Skoog mineral solution; 2,4-D—2,4-dichlorophenoxyacetic acid; Kin—kinetin; Chr.count.—chromosome count; Flow cyt.—flow cytometry; n.t.—not tested. * Culture conditions refers to callus induction phase.

**Table 3:** Summary of the literature data on de novo shoot bud induction from different explant types of *Aesculus* species. Plant growth regulators used for shoot induction, elongation and rooting are given in the table.

Species/ Hybrid	Explant Source	Explant Type	Shoot Induction	Shoot Elongation	Rooting	Reference
*A. carnea*	15-year-old tree	shoot tips	MS + 5 μM BA + 0.1μM IBA	MS + 1 μM BA + 10 μM GA_3_	1/4 WPM + 0.1 μM IBA + 10 g L^−1^ AC	[120]
	40-year-old tree	shoot tips	MS + 2.2 μM BA + 2.3 μM Kin	n.t.	n.t.	[121]
	3 cm-long somatic seedlings	whole seedlings	MS + 10 μM BA	n.t.	n.t.	[87]
*A. hippocastanum*	in vitro plants	stem segments, young leaves, petioles	WPM + 8.9 μM BA + 0.5 μM NAA	WPM + 1.04 μM mT	WPM + 2.7 μM NAA	[119]
	3 cm-long somatic seedlings	whole seedlings	MS +10 μM BA	MS + 1 μM BA + 500 mg L^−1^ PVP	10 mM IBA for 1 min, followed by 1/2 MS + 0.02% AC	[122]

BA—benzyladenine; Kin—kinetin (furfuryl aminopurine); TDZ—thidiazuron; mT—meta-Topolin; IBA—indole-3-butyric acid; NAA—α-naphthaleneacetic acid; IAA—indole-3-acetic acid; GA_3_—gibberellic acid; PVP—polyvinylpyrrolidone; WPM—woody plant medium; AC—activated charcoal; MS—Murashige and Skoog medium; n.t.—not tested.

## Data Availability

Not applicable.
